# Mutations of *NOTCH3* in childhood pulmonary arterial hypertension

**DOI:** 10.1002/mgg3.58

**Published:** 2014-04-01

**Authors:** Ayako Chida, Masaki Shintani, Yoshihisa Matsushita, Hiroki Sato, Takahiro Eitoku, Tomotaka Nakayama, Yoshiyuki Furutani, Emiko Hayama, Yoichi Kawamura, Kei Inai, Shinichi Ohtsuki, Tsutomu Saji, Shigeaki Nonoyama, Toshio Nakanishi

**Affiliations:** 1 Department of Pediatrics, National Defense Medical College3-2 Namiki, Tokorozawa, Saitama, 359-8513, Japan; 2 Department of Pediatric Cardiology, Tokyo Women's Medical University8-1 Kawada-cho, Shinjuku-ku, Tokyo, 162-8666, Japan; 3 Department of Preventive Medicine and Public Health, National Defense Medical College3-2 Namiki, Tokorozawa, Saitama, 359-8513, Japan; 4 Division of Pediatric Cardiology, Department of Pediatrics, Okayama University2-5-1 Shikata-cho, Okayama, 700-8558, Japan; 5 Department of Pediatrics, Toho University Medical Center, Omori Hospital6-11-1 Omori-nishi, Ota-ku, Tokyo, Japan

**Keywords:** ER stress, gene mutation, *NOTCH3*, pulmonary arterial hypertension

## Abstract

Mutations of *BMPR2* and other TGF-*β* superfamily genes have been reported in pulmonary arterial hypertension (PAH). However, 60–90% of idiopathic PAH cases have no mutations in these genes. Recently, the expression of NOTCH3 was shown to be increased in the pulmonary artery smooth muscle cells of PAH patients. We sought to investigate *NOTCH3* and its target genes in PAH patients and clarify the role of NOTCH3 signaling. We screened for mutations in *NOTCH3*, *HES1*, and *HES5* in 41 PAH patients who had no mutations in *BMPR2*, *ALK1*, *endoglin*, *SMAD1/4/8*, *BMPR1B*, or *Caveolin-1*. Two novel missense mutations (c.2519 G>A p.G840E, c.2698 A>C p.T900P) in *NOTCH3* were identified in two PAH patients. We performed functional analysis using stable cell lines expressing either wild-type or mutant NOTCH3. The protein-folding chaperone GRP78/BiP was colocalized with wild-type NOTCH3 in the endoplasmic reticulum, whereas the majority of GRP78/BiP was translocated into the nuclei of cells expressing mutant NOTCH3. Cell proliferation and viability were higher for cells expressing mutant NOTCH3 than for those expressing wild-type NOTCH3. We identified novel *NOTCH3* mutations in PAH patients and revealed that these mutations were involved in cell proliferation and viability. NOTCH3 mutants induced an impairment in NOTCH3-HES5 signaling. The results may contribute to the elucidation of PAH pathogenesis.

## Introduction

Pulmonary arterial hypertension (PAH) is a progressive, severe, potentially fatal disease with an estimated incidence of approximately 1–2 patients per million per year (Gaine and Rubin 1998). Idiopathic PAH (IPAH) is a sporadic form of the disease in which there is neither a family history of PAH nor an identified risk factor (Simonneau et al. 2009). Heritable PAH (HPAH) is inherited in an autosomal dominant fashion with 10–20% penetrance, and affects females approximately twice as often as males (Machado et al. 2009).

Bone morphogenetic protein (BMP) receptor 2 (*BMPR2*), a member of the transforming growth factor (TGF)-*β* superfamily, was identified as a primary gene for HPAH in 2000 (Deng et al. 2000; Lane et al. 2000). Subsequent studies of the TGF-*β* superfamily revealed additional genes responsible for PAH: activin receptor-like kinase 1 (*ALK1*), *endoglin* (*ENG*), *SMAD1/4/8*, and bone morphogenetic protein receptor 1B (*BMPR1B*) (Trembath et al. 2001; Harrison et al. 2003, 2005; Shintani et al. 2009; Chida et al. 2012; Nasim et al. 2012). In addition, Austin et al. (2012) identified *Caveolin-1* (*CAV1*) mutations in PAH patients using whole exome sequencing. These genetic studies have considerably enhanced our understanding of the molecular basis of PAH. However, almost 30% of HPAH cases and 60–90% of IPAH cases have no mutations in *BMPR2*, *ALK1*, *ENG*, *SMAD 1/4/8*, *BMPR1B*, or *CAV1*.

In 2009, Li et al. reported that human pulmonary hypertension could be characterized by the overexpression of NOTCH3 (OMIM 600276) in small pulmonary artery smooth muscle cells (SMCs), and that the severity of the disease in humans and rodents is correlated with the amount of NOTCH3 protein in the lungs (Li et al. 2009). Therefore, we hypothesized that genes belonging to the NOTCH3 signaling pathways, in addition to the BMP signaling pathway, may be associated with the onset of IPAH/HPAH. Accordingly, we screened for mutations in *NOTCH3* and its target genes, *HES1* and *HES5*.

## Materials and Methods

### Subjects

**Figure 1:**
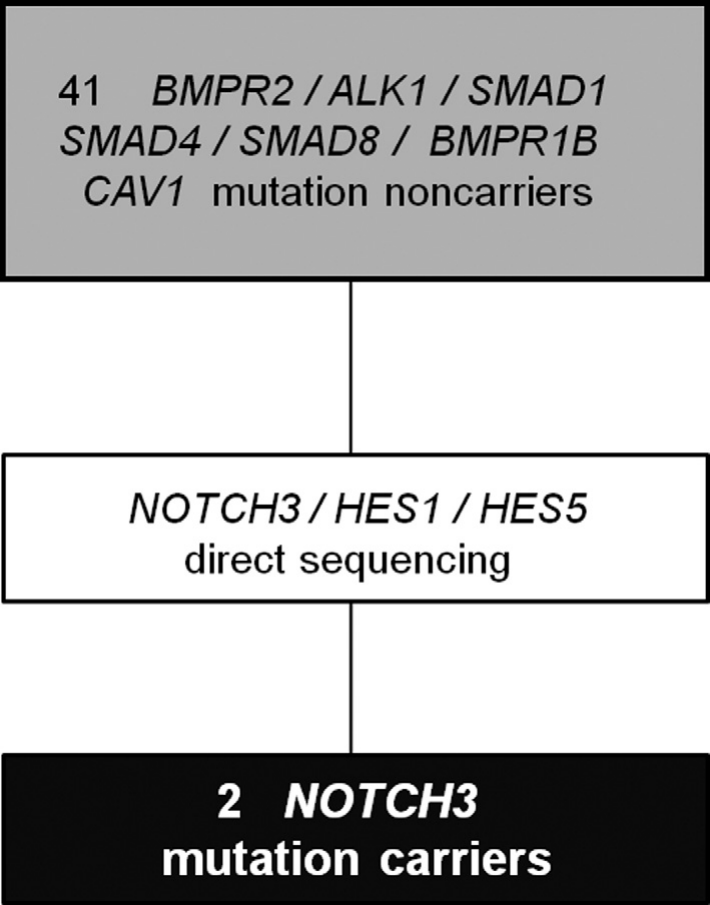
Patient disposition.

From a previous screen of patients with IPAH/HPAH (Shintani et al. 2009; Chida et al. 2012), we identified 41 patients who did not have mutation in *BMPR2*, *ALK1*, *SMAD1*, *SMAD4*, *SMAD8*, *BMPR1B*, or *CAV1*. These patients formed the basis of this study (Fig. 1). The diagnosis of IPAH/HPAH was made through a clinical evaluation, echocardiography, and cardiac catheterization based on the following criteria: mean pulmonary artery pressure >25 mmHg at rest (Galiè et al. 2009). Patients with PAH associated with another disease, such as portal hypertension or congenital heart disease, were excluded from this study by trained cardiologists. This study was approved by the Institutional Review Committee of Tokyo Women's Medical University. Written informed consent was obtained from all patients or their guardians in accordance with the Declaration of Helsinki.

### Molecular analysis

For the 41 patients with no mutations in *BMPR2*, *ALK1*, *SMAD 1/4/8*, *BMPR1B*, or *CAV1*, all coding exons and adjacent intronic regions for *NOTCH3*, *HES1*, and *HES5* were amplified from the genomic DNA using polymerase chain reactions (PCR primer details are available in Table S1). PCR-amplified products were purified and screened via bidirectional direct sequencing with an ABI 3130xl DNA Analyzer (Applied Biosystems, CA). All of the generated sequences were compared with wild-type *NOTCH3* (GenBank NM_000435), *HES1* (GenBank NM_005524), and *HES5* (GenBank NM_001010926).

### Stable cell lines

We established stable HEK293 cell lines in which the expression of NOTCH3 was inducible using the tetracycline regulatory system because it was known that the expression of NOTCH3 in vascular SMCs caused excessive cell death as early as 2 days after transfection (Takahashi et al. 2010). T-REx 293 cells (Invitrogen, Carlsbad, CA) were grown in high glucose DMEM (Sigma, St Louis, MO) supplemented with 10% fetal bovine serum (Gibco, Grand Island, NY), 2 mmol/L l-glutamine, and 5 *μ*g/mL blasticidin (Gibco). The wild-type and mutant NOTCH3 constructs were transfected into T-REx 293 cells using Lipofectamine 2000 reagent (Invitrogen). After 48 h, cells were selected in the presence of 100 *μ*g/mL Zeocin and 5 *μ*g/mL Blasticidin-S (Invitrogen). After treatment with tetracycline (Tet; 2 *μ*g/mL) for 24 h, the expression level of NOTCH3 was determined by western blotting using AbN2. Stable cell lines were maintained in high glucose DMEM-containing 10% fetal bovine serum, 5 *μ*g/mL Zeocin, and 5 *μ*g/mL Blasticidin-S.

### Preparation of plasmids, antibodies, and ligands

Human pcDNA4/TO-NOTCH3 was kindly provided by Dr. Atsushi Watanabe (Aichi, Japan). The reporter gene construct pHes5-luc was a kind gift from Dr. Ryoichiro Kageyama (Kyoto, Japan). Site-directed mutagenesis was carried out using a site-directed mutagenesis kit (Stratagene, CA). The antibodies used were as follows: rabbit polyclonal anti-human NOTCH3 antibodies (AbN2; gifts from A. Watanabe), monoclonal NOTCH3 antibody (3A2; gift from A. Watanabe), anti-*β* actin mouse antibody (Sigma-Aldrich, MO), anti-mouse ERp72 antibody, anti-mouse Calnexin antibody, and anti-mouse BiP/GRP78 antibody (R&D Systems, Oxon, UK). AbN2 and 3A2 have previously been shown to specifically recognize the NOTCH3 protein (Takahashi et al. 2010). Recombinant human Jagged-1 Fc chimera and human BMP4 enzyme-linked immunosorbent assays were from R&D Systems.

### Statistical analysis

Data are presented as means with standard deviation (SD). Differences between the means were evaluated by the Dunnett's test following one-way or two-way analysis of variance (ANOVA). Values of *P* < 0.05 were considered significant. All statistical analyses were performed using SAS version 9.3 (SAS Institute, Cary, NC).

The methods of western blotting and immunoprecipitation, immunocytochemistry, luciferase assay, and cell proliferation and viability tests are detailed in the Data S1.

## Results

### Identification of two novel *NOTCH3* mutations

**Figure 2:**
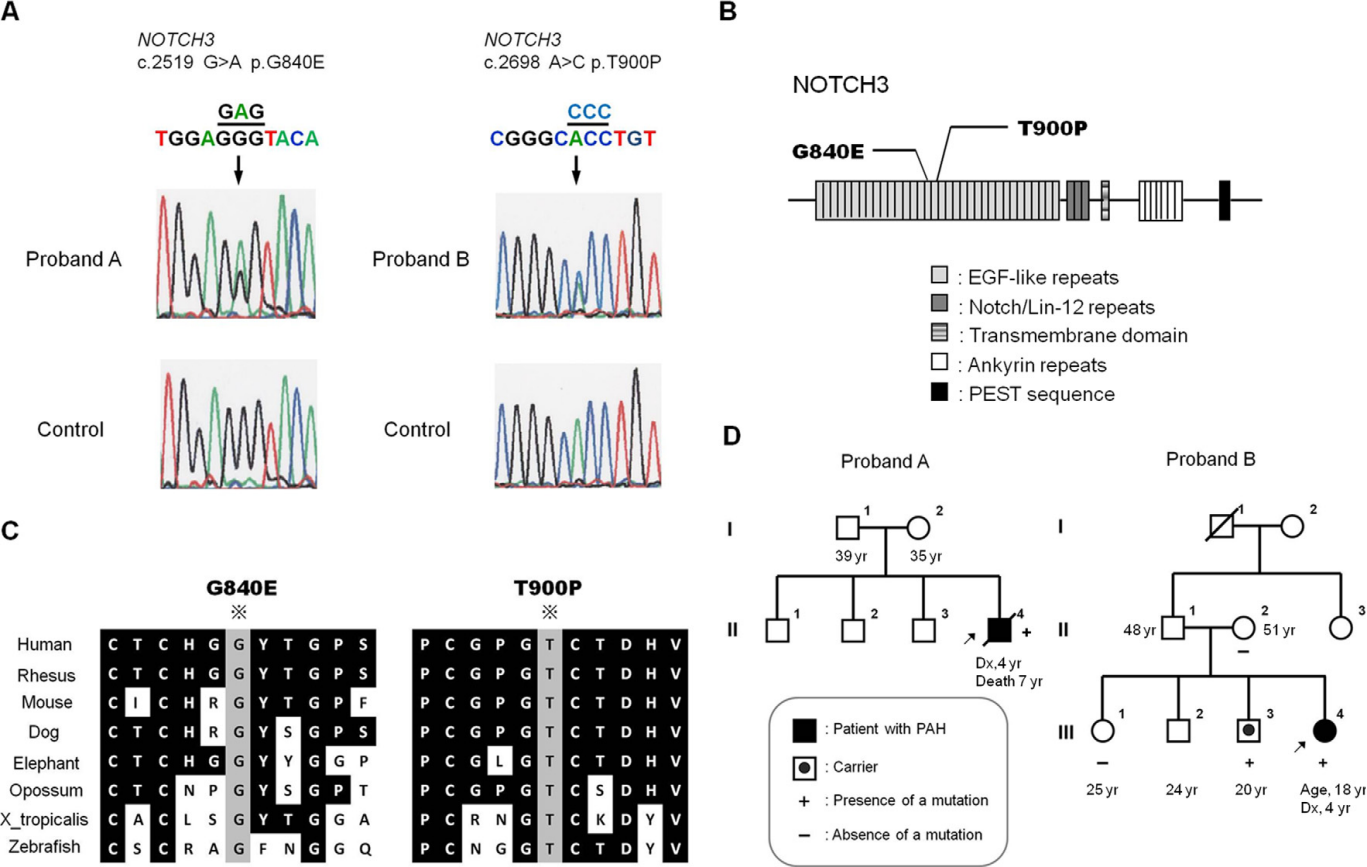
*NOTCH3* mutations in idiopathic pulmonary arterial hypertension patients. (A) Two mutations, c.2519 G>A p.G840E and c.2698 A>C p.T900P, were identified in two probands. (B) Schematic representation of the wild-type NOTCH3 protein and the locations of the two mutations. (C) Alignment of NOTCH3 proteins among human, rhesus monkey, mouse, dog, elephant, opossum, chicken, *Xenupus tropicalis*, and zebrafish, showing the conservation of glycine 840 and threonine 900 in these species. (D) Pedigrees of the patients' families. The current age or the age at diagnosis, as well as the age at diagnosis (Dx) is provided for each family member.

We screened for mutations in the *NOTCH3*, *HES1*, and *HES5* genes in 41 IPAH/HPAH patients who had no mutations in *BMPR2*, *ALK1*, *ENG*, *SMAD1/4/8*, *BMPR1B*, or *CAV1* (Fig. 1). In these 41 patients, the median age at diagnosis was 9 years (range: 0–62 years). The number of male patients was 18 (44 %). Although no mutations were identified in *HES1* or *HES5*, we identified two *NOTCH3* missense mutations in two independent probands with IPAH: a c.2519 G>A p.G840E mutation in proband A, and a c.2698 A>C p.T900P mutation in proband B (Fig. 2A). As depicted in Figure 2B, NOTCH3 consists of 34 epidermal growth factor (EGF)-like repeats, three Notch/Lin-12 repeats, a transmembrane domain, seven ankyrin repeats, and a PEST sequence (rich in proline [P], glutamic acid [E], serine [S], and threonine [T]). Both the G840E and T900P mutations were located in the EGF-like repeats. The alignment of the NOTCH3 protein of nine distantly related species showed that the G840 and T900 amino acids were highly conserved (Fig. 2C). The G840E and T900P mutations were absent in 170 Japanese healthy controls and additional 300 Caucasian healthy controls and were not found in the 1000 Genomes Database (http://www.1000genomes.org) or the Exome Variant Server (EVS) Database (evs.gs.washington.edu/EVS). Besides, in Polyphen-2 (Polymorphism phenotyping v2) (Adzhubei et al. 2010) and the SIFT Human Protein (Kumar et al. 2009), both variants were regarded as “Probably damaging” and “Damaging,” respectively.

In proband A, there was no family history of PAH (Fig. 2D). The subject's family members were not screened for *NOTCH3* mutations because their blood samples could not be obtained. Although there was no family history of PAH in proband B, the same mutation was identified in the patient's youngest elder brother (Figs. 2D and S1). The patient's mother and elder sister did not have the mutation. The other family members of proband B were not screened for *NOTCH3* mutations because their blood samples could not be obtained.

### Clinical characteristics of patients

#### Proband A

When the patient was 4 years old, he visited the clinic because of upper respiratory inflammation. A physical examination revealed a cardiac murmur. Cardiomegaly was identified by a chest X-ray and the patient was diagnosed with IPAH. The hemodynamic data of the patient at 5 years of age revealed a mean pulmonary arterial pressure (mPAP) of 70 mmHg and a right atrial pressure (RAP) of 7 mmHg. The patient's condition progressed to the World Health Organization (WHO) functional class II at 5 years of age. The patient had been administered sildenafil, bosentan, beraprost, and warfarin since the age of 4. When he was 5 years old, the patient began home oxygen therapy (HOT) and diuretics. He did not receive epoprostenol because his family's consent was not obtained. The patient's condition deteriorated despite increases in the doses of sildenafil, bosentan, and veraprost. The patient suffered cardiopulmonary arrest caused by pulmonary hypertension crisis at 6 years old; he was successfully resuscitated and began intravenous epoprostenol therapy. Although the dose of epoprostenol was increased gradually, the patient's heart failure worsened. When he was 7 years old, the patient was discharged from hospital after beginning home intravenous epoprostenol. However, the patient died 9 days later because of a pulmonary hemorrhage.

The patient's past history included brain infarction and right hemiparesis caused by Moyamoya disease. The patient had left cerebral arterial revascularization surgery when he was 3 years old. No complications were reported in the aorta or renal arteries.

#### Proband B

This patient's first symptom was fatigue at 4 years of age. At the initial visit, the patient's right ventricle pressure was almost equal to left ventricle pressure and her brain natriuretic peptide level was 1380 pg/mL. Although the patient was administered beraprost, she became more impaired, and began intravenous epoprostenol at the age of 5. This therapy was initially very effective; however, the patient's mPAP progressively increased. Increasing the amount of epoprostenol and administering sildenafil and bosentan were not effective. The patient's hemodynamic data at 15 years of age revealed an mPAP of 67 mmHg, a RAP of 12 mmHg, a CI of 2.9 L min^−1^ m^−2^, and a pulmonary artery wedge pressure of 12 mmHg. The patient has been receiving intravenous epoprostenol, home oxygen therapy, sildenafil, bosentan, cardiotonic drugs, anticoagulants, and diuretics. The current condition of the patient at 18 years old is WHO functional class III. Currently, the patient and her family do not wish to undergo lung transplantation.

The patient's history includes Graves' disease, which occurred at 13 years old. She initially received thiamazole; however, she stopped that treatment and began potassium iodide because of agranulocytosis. Thyroidectomy was not approved because of severe PH.

### Decreased expression of NOTCH3 and ER-Resident chaperones in mutant cell lines

**Figure 3:**
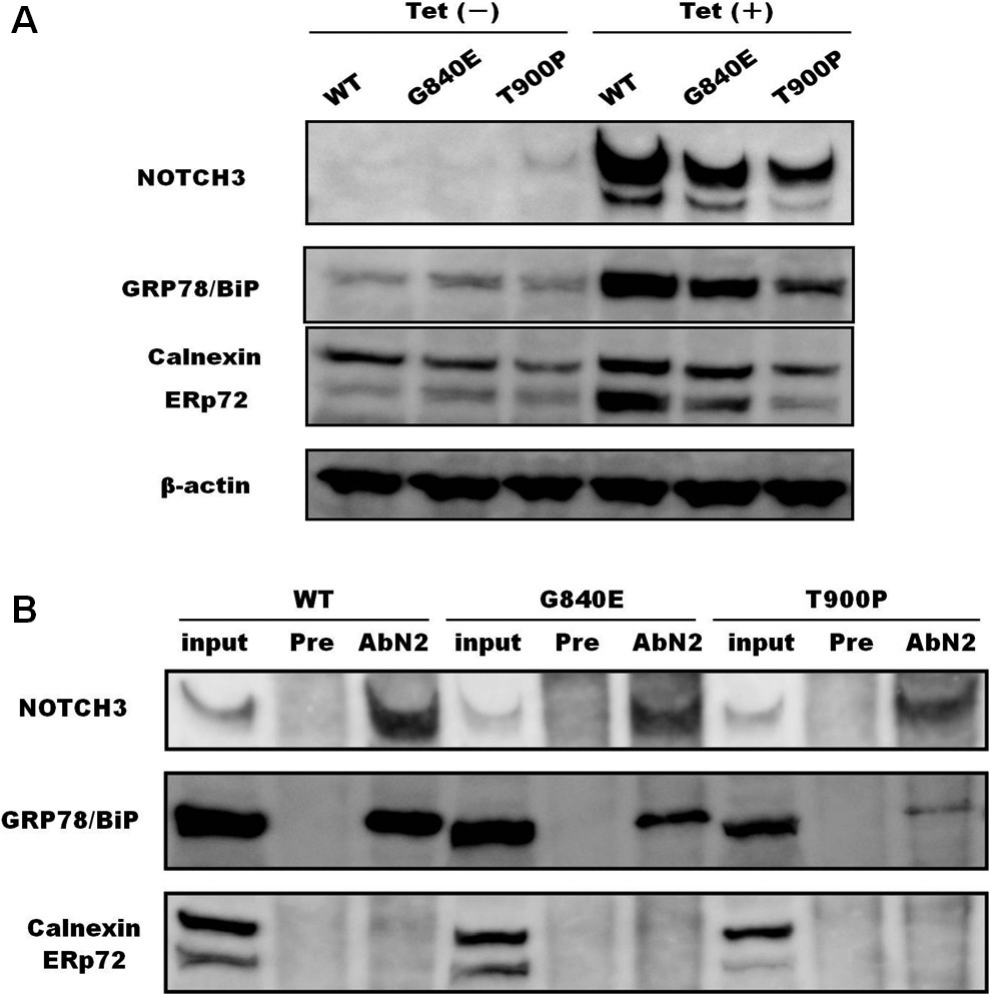
Expression of NOTCH3 and the endoplasmic reticulum (ER) chaperones. (A) Stable cells were incubated with or without 2 *μ*g/mL tetracycline (Tet) for 24 h and these cell lysates (12 *μ*g) were subjected to SDS-gel electrophoresis. We assessed more than three stable cell lines for each of the wild-type and mutant NOTCH3 proteins. The experiment was performed three times. (B) Interaction between ER chaperones and NOTCH3. Cell lysates (200 *μ*g) from stable cell lines were subjected to immunoprecipitation using an anti-NOTCH3 antibody (AbN2) or preimmune rabbit IgG (Pre). Immunoprecipitated complexes were subjected to SDS-PAGE and western blotting. The experiments were performed in triplicate for each stable cell line (WT-17, G840E-36, and T900P-33).

According to a previous report (Takahashi et al. 2010), we established stable HEK293 cell lines in which the expression of NOTCH3 was inducible using the tetracycline-on (Tet-on) regulatory system (T-REx System; Invitrogen). Several cell lines were established, and three cell lines for each construct were selected for NOTCH3 expression experiments. The expression of the mutant versions of NOTCH3 was lower than that of wild-type NOTCH3 (Fig. 3). Additionally, the expression levels of three endoplasmic reticulum (ER)-resident chaperones, GRP78/BiP, Calnexin, and ERp72, were higher in cells overexpressing wild-type NOTCH3 than in cells overexpressing the NOTCH3 mutants, particularly for the T900P-NOTCH3 mutant (Figs. 3A and S2). In Figure S3, the mutant NOTCH3 protein disappeared more quickly than wild-type NOTCH3 protein. Based on the result, the lower expression levels of mutant NOTCH3 in Figure 3A were caused by the high clearance speed.

### NOTCH3 mutants fail to fully bind to GRP78/BiP

On the basis of the findings illustrated in Figure 3A, we investigated whether mutant NOTCH3 interacts with ER chaperones. As shown in Figure 3B, GRP78/BiP coimmunoprecipitated with wild-type NOTCH3, indicating that wild-type NOTCH3 interacted with this ER chaperone. The amount of GRP78/BiP that coprecipitated with the NOTCH3 mutants was markedly lower than the amount that coprecipitated with wild-type NOTCH3. Calnexin and ERp72 were not detected in any of the wild-type or mutant NOTCH3-immunoprecipitated samples.

### Changes in NOTCH3 levels and GRP78/BiP localization

**Figure 4:**
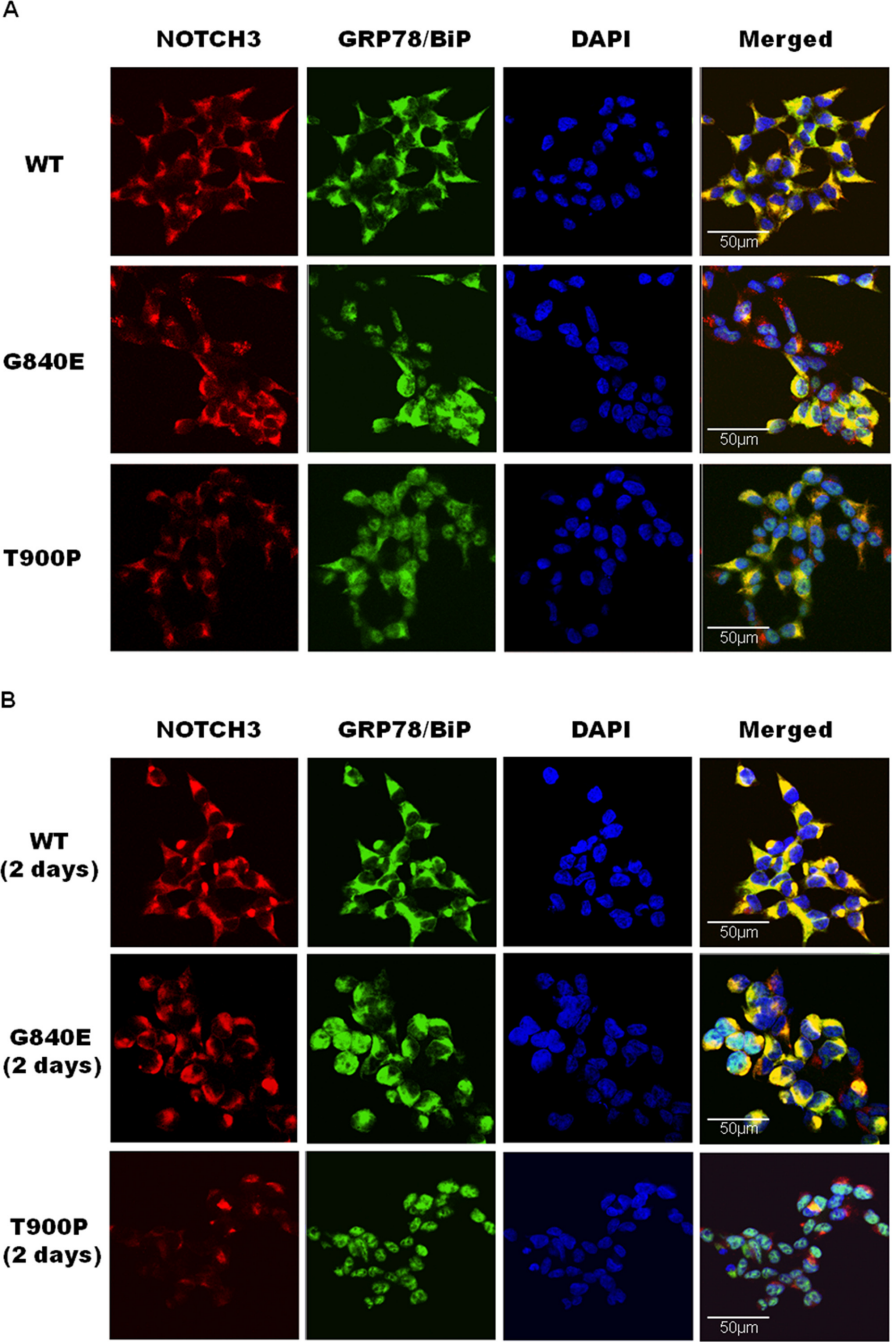
Changes in the quantity of NOTCH3 aggregates and the localization of GRP78/BiP. (A) Nine stable cell lines (WT-17, WT-21, WT-38, G840E-5, G840E-21, G840E-36, T900P-14, T900P-28, and T900P-33) were treated with 2 *μ*g/mL tetracycline for 24 h and double-stained with an anti-Notch3 antibody (AbN2) and a GRP78/BiP antibody. NOTCH3 (red) was detected by an Alexa Fluor 568-labeled secondary antibody and GRP78/BiP (green) was detected by an Alexa Fluor 488-labeled secondary antibody. (B) Stable cells were treated with tetracycline for 24 h and then incubated without tetracycline for 2 days. Cells were double-stained as described above.

We examined the quantity and localization of wild-type or mutant NOTCH3 via immunostaining using the ER marker GRP78/BiP. While many wild-type NOTCH3 aggregates were detected in the perinuclear region of the cytoplasm, very few mutant NOTCH3 perinuclear aggregates were detected, particularly in cells transfected with T900P-NOTCH3 (Fig. 4A). Aggregates were colocalized with GRP78/BiP in cells expressing wild-type NOTCH3. However, in cells expressing G840E-NOTCH3, GRP78/BiP was partly localized in the nucleus and did not fully colocalize with NOTCH3 aggregates. This was particularly evident in cells expressing T900P-NOTCH3. When present in the nucleus, GRP78/BiP was colocalized with nuclear bodies (Fig. 4A).

### Clearance of NOTCH3 aggregates

We next examined the degradation of wild-type and mutant NOTCH3 aggregates after 2 days using Tet-on cells. Figure 4B shows that wild-type NOTCH3 and G840E-NOTCH3 aggregates degraded slowly, whereas T900P-NOTCH3 aggregates disappeared rapidly. As was the case for stable cells immediately after Tet-on, GRP78/BiP was located in the nucleus in cells expressing mutant NOTCH3, most notably in cells expressing T900P-NOTCH3. These findings were confirmed by western blotting of cells expressing NOTCH3 (Fig. S3).

### Promotion of cell proliferation and viability by mutant NOTCH3 expression

**Figure 5:**
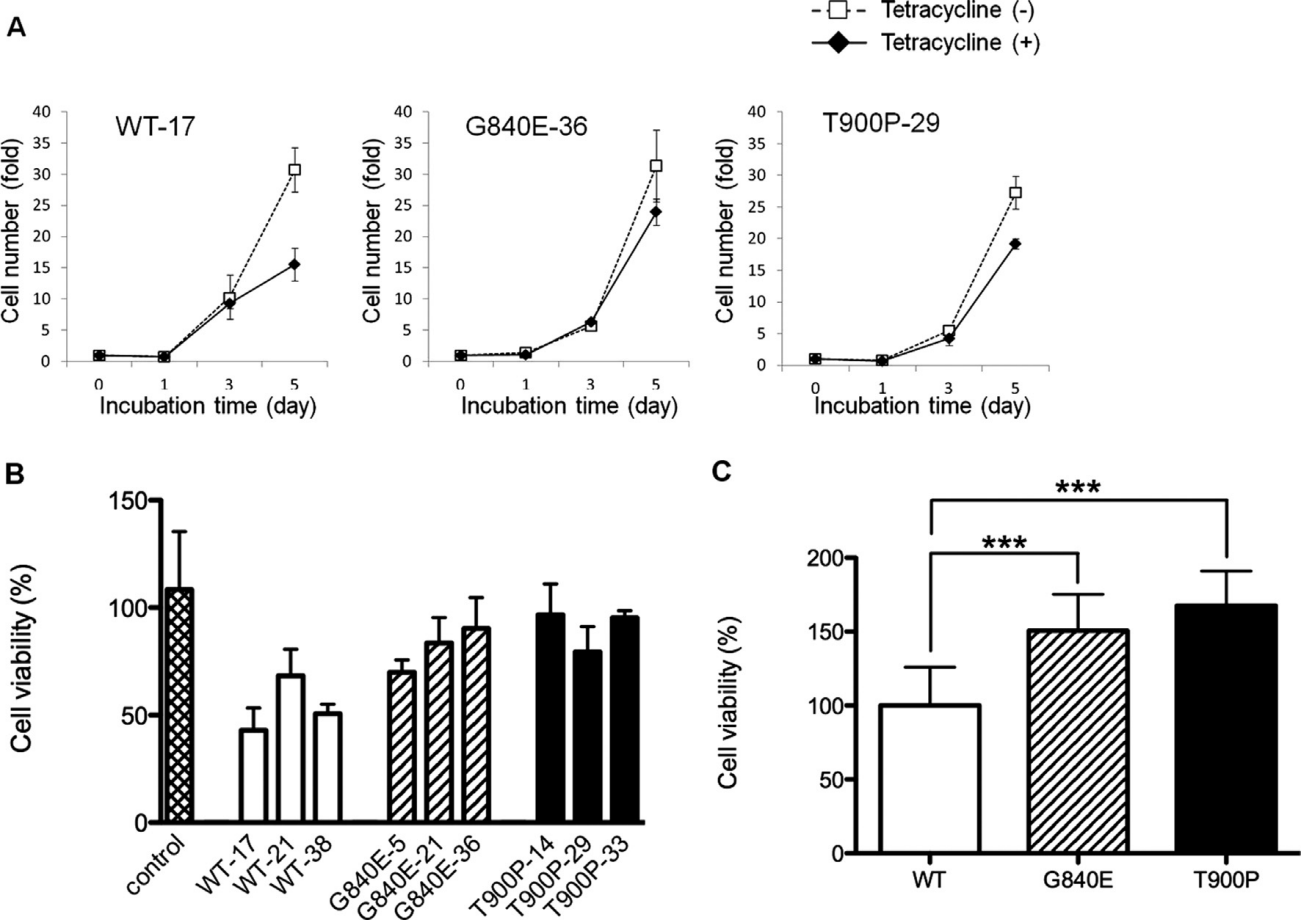
Cell proliferation and viability of NOTCH3. (A) Stable cells were incubated with (closed argyles) or without (open squares) tetracycline and harvested on days 0, 1, 3, and 5 for each of the nine stable cell lines (WT-17, WT-21, WT-38, G840E-5, G840E-21, G840E-36, T900P-14, T900P-29, and T900P-33). Each data point represents the mean number of cells with the standard deviation from four independent experiments. (B) Stable cells were incubated with or without tetracycline for 3 days. Cell viability was determined using the WST-1 reagent. The control indicates T-REx 293 cells that were not transfected. Values represent the means with the standard deviation from three independent experiments and are shown as the percentage of the total number of cells without tetracycline treatment. (C) The cell viability demonstrated in (B) was converted to a percentage of the mean value relative to that of stable cells expressing wild-type NOTCH3. Each data point represents the mean and standard deviation from nine cell lines. ***P < 0.001.

Cell proliferation was assessed by cell counting at 1 day, 3 days, and 5 days after Tet-on. The expression of wild-type NOTCH3 in the Tet-on cells caused a decrease in cell number compared to the untreated cells; however, the expression of mutant NOTCH3 had little impact on cell growth (Fig. 5A). We evaluated the ratio of the number of Tet-on cells to that of untreated cells on day 5 (Fig. 5A). The ratio of G840E-36 (0.78 ± 0.05) and T900P (0.70 ± 0.10) were significantly higher than that of WT-17 (0.50 ± 0.04, *P* = 0.004 and 0.021, respectively). These findings were confirmed via a cell proliferation assay using the WST-1 reagent (Fig. 5B and 5C). While the cells expressing wild-type NOTCH3 showed viability that was equivalent to that of untreated cells, the cells expressing mutant NOTCH3 exhibited a significantly higher level of viability. These cells reached confluence and did not show any detectable apoptosis or morphological abnormalities.

### The NOTCH3 mutant induces the downregulation of NOTCH3-HES5 signaling activity

**Figure 6:**
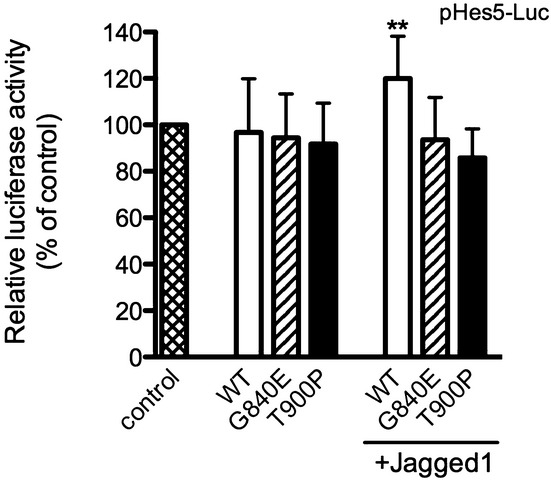
Luciferase activity induced by *NOTCH3*. T-REx 293 cells were transfected with pHes5-Luc and any one of wild-type NOTCH3, mutant NOTCH3, or the empty pcDNA4/TO vector. Some of the cells were cultured with preclustered Jagged-1 Fc (100 ng/mL) for 4 h. Values represent the mean with standard deviation of four independent experiments. The means were compared with wild-type (WT without stimulation with Jagged-1) using one-way ANOVA followed by Dunnett's test. **P < 0.01.

We investigated the transcriptional activity mediated by wild-type or mutant NOTCH3 with or without Jagged-1 to determine whether mutant NOTCH3 could increase or decrease NOTCH3-responsive target gene activity. To this end, we used a pHes5-luc reporter, where the luciferase Hes5 promoter drives expression to assess NOTCH3 activity. The luciferase assay showed that wild-type NOTCH3 exhibited significantly higher luciferase activity with Jagged-1 stimulation than without Jagged-1 stimulation. After stimulation with Jagged-1, however, G840E-NOTCH3 and T900P-NOTCH3 did not induce higher activity than mutant NOTCH3 without Jagged-1 stimulation (Fig. 6). These findings indicated that NOTCH3 mutants induced an impairment in NOTCH3-HES5 signaling.

## Discussion

This is the first study to report the identification of two missense mutations in *NOTCH3* in IPAH patients. Both of the mutations identified in this study result in amino acid substitutions in the highly conserved and functionally essential EGF-like domain, and both mutations were absent in 170 Japanese and 300 Caucasian healthy controls, the 1000 Genomes Database, and the EVS Database.

*NOTCH* genes encode a group of 300-kD single-pass transmembrane receptors (*NOTCH1–4*). The large extracellular domain contains tandem EGF-like repeats and cysteine-rich Notch/LIN-12 repeats, and Ankyrin repeats and a PEST sequence have been found within the intracellular domain (ICD) (Artavanis-Tsakonas et al. 1999). The NOTCH signaling pathway is highly evolutionarily conserved and is critical for cell fate determination during embryonic development, including many aspects of vascular development (Wang et al. 2008). Upon interacting with its ligands (Jagged-1, Jagged-2, and Delta family) expressed on neighboring cells, NOTCH undergoes proteolytic cleavage, which frees the ICD from the plasma membrane (Xia et al. 2012). This change induces translocation of the ICD into the nucleus. The ICD then forms a complex with the transcriptional repressor CBF1/RBP-jk, which induces the transcription of downstream target genes, including *HES1/5/7* and the *HEY* family genes (Iso et al. 2003; Weber 2008).

NOTCH3 is predominantly expressed in arterial vascular SMCs, where it regulates SMC differentiation (Zhu et al. 2011; Xia et al. 2012). The main NOTCH3 ligand is Jagged-1, which is also expressed in arterial endothelial cells (ECs) (Xia et al. 2012). Taking these reports into consideration, we decided to perform functional analysis of mutant NOTCH3 through the use of Jagged-1 Fc.

*NOTCH3* mutations are known to be associated with cerebral autosomal dominant arteriopathy with subcortical infarcts and leukoencephalopathy (CADASIL) (Joutel et al. 1996). More than 150 *NOTCH3* mutations have been reported in CADASIL patients; mutation hot spots are located in exons 3 and 4 (Chabriat et al. 2009). Both of the *NOTCH3* mutations that we identified in IPAH patients were missense mutations located in exons 16 and 17. To date, no mutations in these two exons have been reported in CADASIL patients, and there have been no reports of a PAH complication in CADASIL.

In this study, we revealed that compared to the wild-type protein, mutant NOTCH3 formed aggregates poorly and decreased the amount of GRP78/BiP, Calnexin, and ERP72. Our immunoprecipitation studies suggested that GRP78/BiP cannot interact with mutant NOTCH3. In addition, we found that T900P-NOTCH3 was degraded more rapidly than wild-type NOTCH3. These findings are not consistent with a previous report that investigated CADASIL mutations using similar methods (Takahashi et al. 2010); however, these differences may be attributable to the difference in the location of the mutations.

In an immunocytochemistry study, we indicated that GRP78/BiP and wild-type NOTCH3 were colocalized in the ER, and that GRP78/BiP was localized in the nuclei of cells expressing mutant NOTCH3. GRP78, a member of the HSP70 gene family, has been traditionally regarded as a major ER chaperone that facilitates protein folding and assembly, protein quality control, Ca^2+^ binding, and the regulation of ER stress signaling (Ni et al. 2011). It is also known that GRP78 can be observed in the nucleus when it is ectopically overexpressed or induced by ER stress (Reddy et al. 2003; Huang et al. 2009). On the basis of this information, we hypothesize that the mutant NOTCH3 protein induces a high level of stress on the ER and induces GRP78/BiP nuclear translocation. Because the mutant NOTCH3 protein is located in the ER, it may not be able to interact with GRP78/BiP in the nucleus. Gene mutations can cause aberrant protein folding and the accumulation of the mutant protein in the ER (Takahashi et al. 2010). Because ER chaperones facilitate ER-associated protein degradation to clear aggregated, misfolded, or unassembled proteins (Ni and Lee 2007), T900P-NOTCH3 proteins may be degraded more rapidly than wild-type NOTCH3.

The cells expressing mutant NOTCH3 showed higher levels of viability and proliferation than the cells expressing wild-type NOTCH3, and did not exhibit apoptosis or morphological abnormalities. These findings suggest that when the normal NOTCH3 protein is absent, increases in cell proliferation rates occur. This result is contrary to the work of Li et al. and taken together the role of NOTCH3 may be more complex than first described. Thus more work is required to resolve this discrepancy.

In the luciferase assay, we showed that the *NOTCH3* mutations we identified caused an impairment in NOTCH3-HES5 signaling activity. It remains unclear whether the *NOTCH3* mutations identified in this study affect NOTCH signaling activity. One study revealed that a part of the mutations identified in CADASIL patients caused a gain in function in the NOTCH signaling pathway (Joutel et al. 2004). Another report indicated that *NOTCH3* mutations in or near the ligand-binding site (EGF-like repeats 10-11, corresponding to a part of exon 7-9) impaired ligand binding sufficiently to affect signaling activity (Peters et al. 2004). Furthermore, another two studies suggested that most of the mutations located outside of the ligand-binding site did not impair the signal transduction activity of NOTCH3 (Low et al. 2006; Monet-Leprêtre et al. 2009). The difference in function between exon 16–17, in which the mutations we identified were located, and other exons corresponding to EGF-like repeats remain unexplained. Further investigations are necessary.

It appears that epoprostenol may not be sufficiently effective in the two PAH patients with *NOTCH3* mutations. Although the reason remains unclear, epoprostenol might not influence to NOTCH signaling. In addition, their age at diagnosis was very young comparatively. It was difficult to identify other additional phenotypic features in the two subjects. The youngest elder brother of proband B has the same *NOTCH3* mutation, but has not exhibited any clinical signs of PAH to date. The *NOTCH3* mutation may have very low penetrance in familial PAH, as well as *BMPR2* mutation in familial PAH. In CADASIL patients, the penetrance of *NOTCH3* mutation is complete by the end of fourth decade (Ayata 2010). Although we cannot clarify whether the youngest elder brother of proband B will develop PAH in the future, it will be necessary to check his health condition periodically.

We identified several limitations of our study. For the *NOTCH3* mutations we identified, a detailed investigation in a larger number of subjects is needed, and a search for mutations of other NOTCH3 pathway genes may also be beneficial. Moreover, although we demonstrated that the mutant NOTCH3 reduced the amount of GRP78/BiP, Dromparis et al. (2013) reported that the expression of GRP78 was increased in chronic normobaric hypoxia-pulmonary hypertension mice. These differences may be a result of the different types of mutations, cells, or species used in the various studies. We acknowledge that the HEK293 cells we used are not the ideal cell lines for functional studies of PAH; thus, it is difficult to prove definitively that NOTCH3 causes PAH based on our findings. However, in the study conducted by Dromparis et al. (2013), it seems that GRP78 was translocated to the nucleus in the pulmonary artery SMCs of hypoxia mice more than normoxia mice. Because this finding is consistent with our immunostaining results, the study supports our hypothesis that mutant NOTCH3 induces a high degree of ER stress. Therefore, additional investigations are necessary to further analyze the function of *NOTCH3* in the pathogenesis of PAH. These studies might include cell proliferation and viability analyses using both ECs and SMCs from human pulmonary arteries and animal models with the *NOTCH3* mutation.

## Supporting Information

**Data S1.** Supplementary Methods **Table S1.** Primer pairs used to amplify the *NOTCH3*, *HES1*, and *HES5* coding sequences. **Figure S1.** Sequence analysis of the *NOTCH3* mutation in the family of proband B. **Figure S2.** Mean ER chaperone/β-actin ratios from densitometry analysis of data presented in Figure 3. The levels of all three chaperones were significantly decreased in cells expressing T900P-NOTCH3 compared with those expressing wild-type NOTCH3 (WT). **P* < 0.05. **Figure S3.** Western blotting of *NOTCH3* degradation. Stable cells were treated with (Tet+) or without (Tet−) tetracycline (2 μg/mL) for 24 h and then incubated in medium without tetracycline. Cells were harvested on days 0, 1, and 2 and were subjected to SDS-PAGE and western blot analysis. The data are representative of experiments performed for three stable cell lines (WT-17, G840E-36, and T900P-33). Experiments were performed twice for each stable cell line.
