# Supplementary material for: Mutations of NOTCH3 in childhood pulmonary arterial hypertension
Source: Mol Genet Genomic Med. 2014 Apr 1;2(3):229–39. doi: 10.1002/mgg3.58 (PMC4049363; doi:10.1002/mgg3.58)
Supplement: Supplementary file 1 [file mgg30002-0229-SD1.doc]

**SUPPLEMENTARY METHODS**

**Western Blotting and Immunoprecipitation**

For the experiments on NOTCH3 activation and gene expression, some of the transfected cells were cultured with preclustered Jagged-1 Fc for 4 h. Preclustering of Jagged-1 Fc was performed by incubating Jagged1-Fc and the anti-Fc antibody (Jackson ImmunoResearch Laboratories, PA) at a 1:1 ratio at 4°C for 20 h. Twenty-two hours after transfection, cells were lysed in Solution A (1 M Tris-HCl [pH 8.0], 50 mM, 0.5 M EDTA 1 mM [pH 8.0], 5 M NaCl 120 mM, 0.25% NP-40, 1% Triton X-100). For western blotting, the lysates were separated on 10% resolving SDS-polyacrylamide gels, and proteins were transferred to polyvinylidene fluoride membranes by semi-dry blotting. The membranes were blocked in TBS-T (50 mM Tris-HCl [pH 7.6], 137 mM NaCl, 0.1% [w/v] Tween 20) containing 1% bovine serum albumin (BSA) for 1 h at room temperature. The membranes were rinsed with TBS-T and incubated with the primary antibody against NOTCH3 (100 ng/mL), Calnexin (1:10,000), ERp72 (1:10,000), BiP/GRP78 (1:10,000), and β-actin (1:10,000) for 1 h at room temperature. The membranes were rinsed with TBS-T and incubated with HRP-goat anti-rabbit IgG (Invitrogen) for NOTCH3 detection, or with anti-mouse IgG (Invitrogen) for Calnexin, ERp72, Bip/GRP78, and β-actin detection. Blots were then washed with TBS-T and bound complexes were detected using enhanced chemiluminescence (ImageQuant LAS 4000 mini, GE Healthcare). Lysates (200 µg of protein) were incubated with Dynabeads Protein G (Invitrogen) and AbN2 antibodies (1 µg/µL) and immunoblotted with the monoclonal NOTCH3 antibody 3A2 for the co-immunoprecipitation assays.

**Immunocytochemistry**

The transfected T-REx 293 cells grown on glass coverslips with poly-l-lysine were washed with phosphate-buffered saline (PBS) 48 h after transfection. Cells were then fixed for 15 min at room temperature in PBS containing 4% paraformaldehyde and washed two times with PBS. Cells were incubated in 3% BSA in TBS-T for 30 min at room temperature to block nonspecific binding. After blocking, cells were incubated with AbN2 (1:1000) and GRP78/BiP (1:400) antibodies at room temperature for 1 h. After washing, detection was achieved by incubation with Alexa Fluor 568 goat anti-rabbit IgG (Invitrogen; 1:1,000) and Alexa Fluor 488 goat anti-mouse IgG (Invitrogen; 1:1000). Cells were mounted with Prolong Gold antifade reagent with DAPI (Invitrogen). Fluorescence digital images were recorded with an LSM 5 PASCAL Laser Scanning Microscope (Carl Zeiss, NY).

**Luciferase assay**

T-REx 293 cells were transfected using Lipofectamine 2000 reagent with pHes5-Luc and any one of wild-type NOTCH3, mutant NOTCH3, or the empty pcDNA4/TO vector. Some of the cells were cultured with preclustered Jagged-1 Fc for 4 h. Cells were harvested 32 hours after transfection. Firefly and renilla luciferase activities were measured with the Dual luciferase reporter assay (Promega, WI) following the manufacturer’s instructions. Results are expressed as the ratio of firefly luciferase activity to renilla luciferase activity. All assays were performed four times.

**Comparison of Cell Proliferation Rates**

Stable cells (2 × 10^4^) were incubated in 24-well plates and incubated with or without 2 µg/mL tetracycline for the indicated times at 0, 1, 3, and 5 days. To determine the number of proliferating cells at each time point, we separately harvested cells from three wells. Cells were stained with trypan blue and counted four times. Data were obtained from three independent experiments.

**Measurement of Cell Viability**

Cell viability was determined by means of a cell proliferation assay using the WST-1 reagent (Roche, Basel, Switzerland). This assay is based on the cleavage of the tetrazolium salt WST-1 to formazan by cellular mitochondrial dehydrogenases.

**SUPPLEMENTARY DATA**

**Table A. Primer pairs used to amplify the *NOTCH3, HES1,* and *HES5* coding sequences.**

NOTCH3

| Exon | Forward Primer | Reverse Primer |
| --- | --- | --- |
| Exon 1 | cgcgtctcactgcatgctc | gctcaagggtccctgttcc |
| Exon 2 | cttcccctgctttgtggttc | tggggaaacacgagaggttg |
| Exon 3, Exon 4 | gtttcttgcctgtcttgtgtgtatc | cacgttcacttcacaattctgacc |
| Exon 5, Exon 6 | gccctactcaggagagtcagag | atccatggctccctgcaga |
| Exon 7, Exon 8 | cagagcaggaagatctgcct | aagggtcccactccaaacc |
| Exon 9, Exon 10 | gcaccccgttcacaccatag | ggtcctgccttgctacaacc |
| Exon 11, Exon 12 | tcagatagagctgaaccaggattg | gacttcaccctcgatctaaggac |
| Exon 13, Exon 14 | gtccctgctgactttgttctg | gatacccacctcccaagctc |
| Exon 15 | atttccctccaggagcttgg | cagcatcatccctgatagggt |
| Exon 16 | agacacgaatgacagcacgg | atgactgtgttccccagagc |
| Exon 17 | taatgggggcaaggtaggtg | aagccagagtccctgctct |
| Exon 18, Exon 19 | taacagcgggactcaggaag | cattcggctcacactagcag |
| Exon 20 | tgtgtgatggaggcagaagg | cccacagatacaccaagagtca |
| Exon 21, Exon 22 | gggttctttgcgtcttcatgg | catgtagatcagccacaatggg |
| Exon 23 | tcattcccccattgtggctg | ccctactcctcctccaaagg |
| Exon 24-1 | tcttttccccactcctccatt | cagcggctgttgttgaagag |
| Exon 24-2 | tgtctccacgggggctcc | gatgaacagacacacggacaga |
| Exon 25 | tgcatggaaggggattgtctc | agacctggatcaacagcatctc |
| Exon 26 | gacctgtgggtggagatgg | gctcccctaagagcaggaag |
| Exon 27 | tggacttcccgtacccact | ggtccagggttcacaaggtc |
| Exon 28 | ccagctggacacctctagtg | agaagaaagggttgcaaagctg |
| Exon 29 | attagctggacaccgtggca | gacctttgtcacttccaaccaag |
| Exon 30 | agctcctaatggggtaccct | caaacccaggtaagtctaatgcct |
| Exon 31 | tgctcctgattcctctgttcc | gttatgtgtgcgtgagcttcag |
| Exon 32 | tggatccagacacaagtacttgg | gggcccccaaaaactttagtc |
| Exon 33-1 | agaggctggaagactttgctac | ctgcatagggcccctcaag |
| Exon 33-2 | cagtcggggtccaagaagag | ggatggggtcaggtaaggg |
| Exon 33-3 | ggctgtgcccctcgattg | caggagggttggtggaaagag |

HES1

| Exon | Forward Primer | Reverse Primer |
| --- | --- | --- |
| Exon 1, Exon2 | gcacttgctcagtagttttgtga | ctgggagaacgcagtaccag |
| Exon 3 | cactctgaagcagctgacac | ctgaatgcctctcacaaccac |
| Exon 4-1 | caacgctagtgtggagaggt | gggaatgaggaaagcaaactgg |
| Exon 4-2 | ccagatcaatgccatgacctac | tagagtccggagggaagaga |

HES5

| Exon | Forward Primer | Reverse Primer |
| --- | --- | --- |
| Exon 1, Exon2 | ccgcgcctatatagggcgt | tagtcctggtgcaggctcttg |
| Exon 3 | cctgaagcacagcaaaggtga | attgtcctaaaacggcagggac |

**
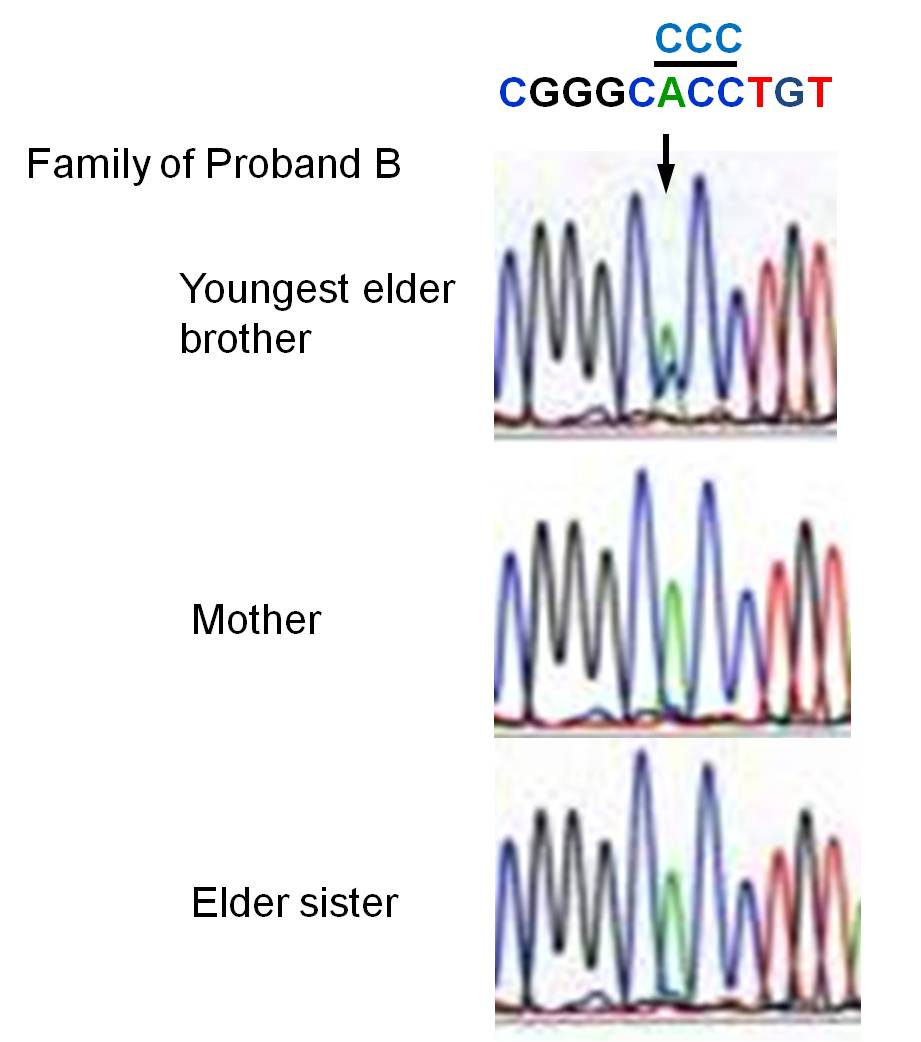
**

**Supplementary Figure S1. Sequence analysis of the *NOTCH3* mutation in the family of proband B.**

**
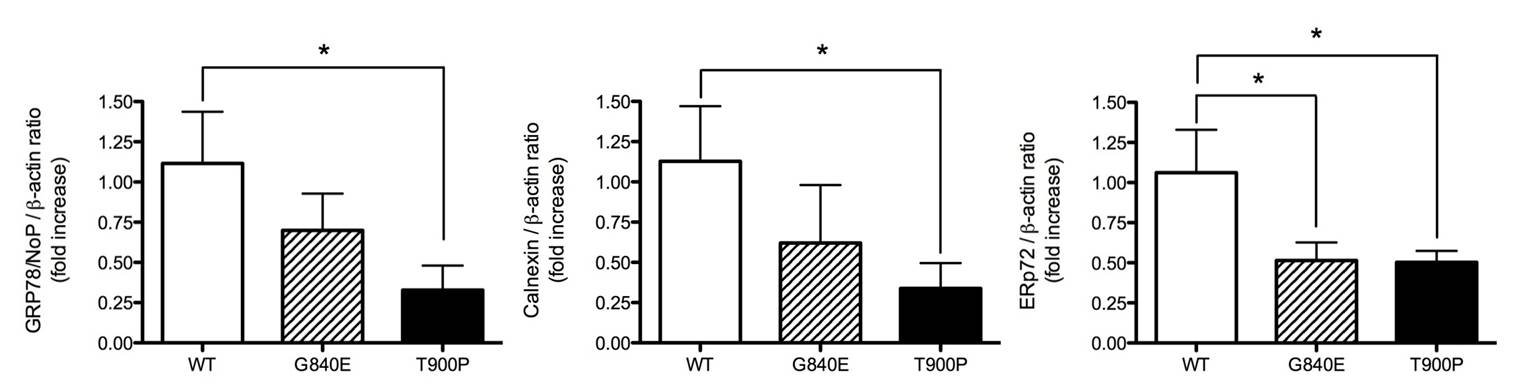
**

**Supplementary Figure S2**. **Mean ER chaperone/β-actin ratios from densitometry analysis of data presented in Figure 3.** The levels of all three chaperones were significantly decreased in cells expressing T900P-NOTCH3 compared with those expressing wild-type NOTCH3 (WT). *p < 0.05.

**
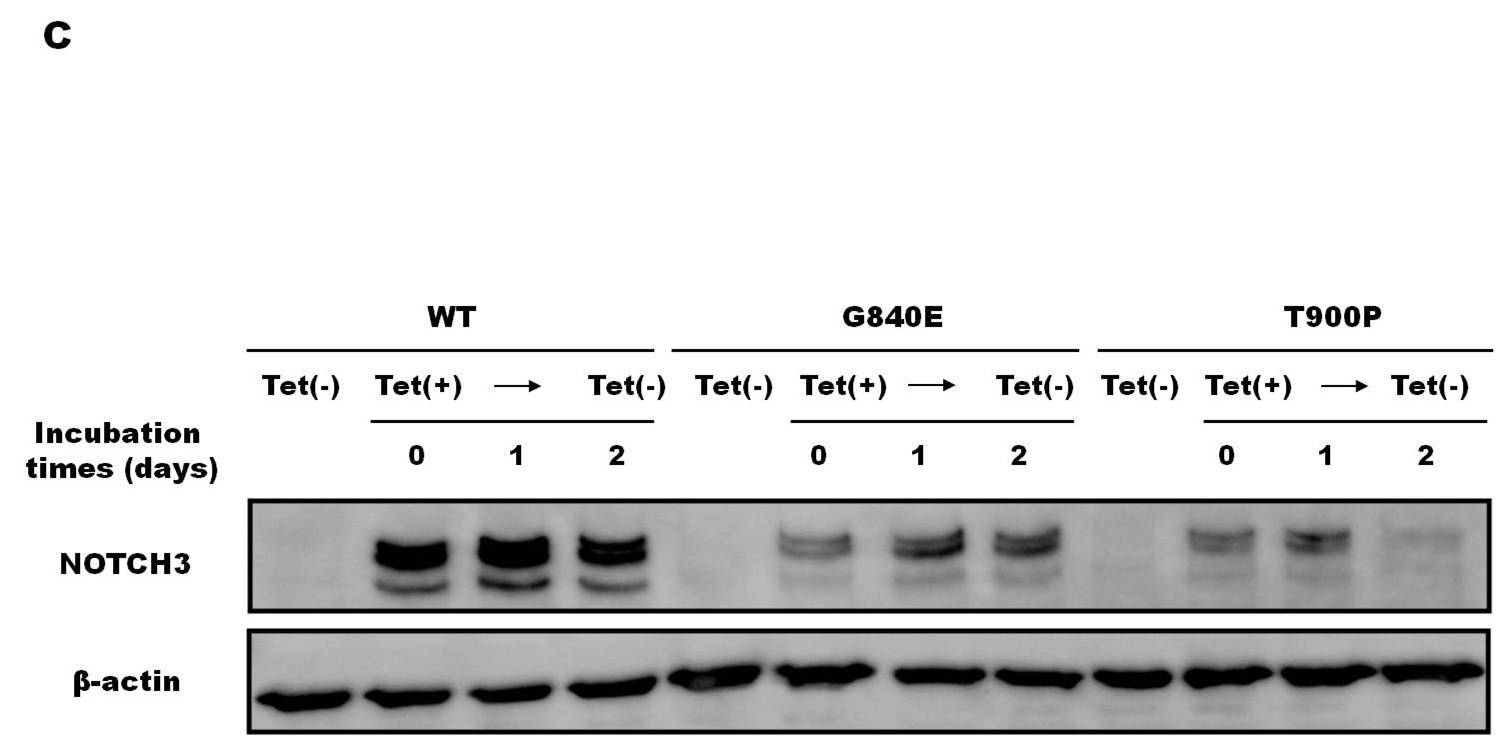
**

**Supplementary Figure S3. Western blotting of NOTCH3 degradation.** Stable cells were treated with (Tet+) or without (Tet-) tetracycline (2 µg/mL) for 24 h and then incubated in medium without tetracycline. Cells were harvested on days 0, 1, and 2 and were subjected to SDS-PAGE and western blot analysis. The data are representative of experiments performed for 3 stable cell lines (WT-17, G840E-36, and T900P-33). Experiments were performed twice for each stable cell line.
